# Discovery and Development of Small Molecule Allosteric Modulators of Glycoprotein Hormone Receptors

**DOI:** 10.3389/fendo.2015.00142

**Published:** 2015-09-14

**Authors:** Selvaraj G. Nataraja, Henry N. Yu, Stephen S. Palmer

**Affiliations:** ^1^TocopheRx Inc., Burlington, MA, USA; ^2^EMD Serono Research and Development Institute Inc., Billerica, MA, USA

**Keywords:** small molecule allosteric modulators, follicle-stimulating hormone, leutinizing hormone/chorionic gonadotropin, thyroid-stimulating hormone, G protein-coupled receptor, glycoprotein hormone receptors, leucine-rich repeat

## Abstract

Glycoprotein hormones, follicle-stimulating hormone (FSH), luteinizing hormone (LH), and thyroid-stimulating hormone (TSH) are heterodimeric proteins with a common α-subunit and hormone-specific β-subunit. These hormones are dominant regulators of reproduction and metabolic processes. Receptors for the glycoprotein hormones belong to the family of G protein-coupled receptors. FSH receptor (FSHR) and LH receptor are primarily expressed in somatic cells in ovary and testis to promote egg and sperm production in women and men, respectively. TSH receptor is expressed in thyroid cells and regulates the secretion of T3 and T4. Glycoprotein hormones bind to the large extracellular domain of the receptor and cause a conformational change in the receptor that leads to activation of more than one intracellular signaling pathway. Several small molecules have been described to activate/inhibit glycoprotein hormone receptors through allosteric sites of the receptor. Small molecule allosteric modulators have the potential to be administered orally to patients, thus improving the convenience of treatment. It has been a challenge to develop a small molecule allosteric agonist for glycoprotein hormones that can mimic the agonistic effects of the large natural ligand to activate similar signaling pathways. However, in the past few years, there have been several promising reports describing distinct chemical series with improved potency in preclinical models. In parallel, proposal of new structural model for FSHR and *in silico* docking studies of small molecule ligands to glycoprotein hormone receptors provide a giant leap on the understanding of the mechanism of action of the natural ligands and new chemical entities on the receptors. This review will focus on the current status of small molecule allosteric modulators of glycoprotein hormone receptors, their effects on common signaling pathways in cells, their utility for clinical application as demonstrated in preclinical models, and use of these molecules as novel tools to dissect the molecular signaling pathways of these receptors.

## Introduction

Glycoprotein hormones, FSH, LH, and TSH, are secreted from the anterior pituitary gland (1, 2). These hormones are composed of two subunits, a common α-subunit and a hormone-specific β-subunit (2, 3). Specificity of the hormone for receptor binding is determined by the β-subunit (4, 5). Chorionic gonadotropin (CG), a homolog of LH, is secreted from the placenta of primates during pregnancy (6, 7). Human CG β-subunit (beta-hCG) gene has evolved from LH β-subunit by gene duplication and reading through into the 3′ untranslated region (8–10). Beta-hCG differs from LH β-subunit at the C-terminal end of the protein, which contains additional 34 amino acids called the C-terminal peptide (CTP) (11). Glycoprotein hormones are characterized by glycosylation of both subunits (11, 12). The common α-subunit carries two N-linked glycans and the β-subunits of all three glycoproteins have one or two N-linked glycans (13). Human CGβ has additional four O-linked glycans in their CTP (14). The N-linked oligosaccharide chains have a minor role in receptor binding of glycoprotein hormones, but they are critical for bioactivity (15). Glycoprotein hormones lacking N-linked oligosaccharides behave as antagonists (16–19). On the other hand, it was suggested that the four O-linked oligosaccharides play an important role in the survival of hCG in circulation, and thus increasing the half-life of the protein (15, 20). Naturally occurring glycosylation variants of hFSH and hCG with differing activity in human granulosa cells have been described (14, 21–23).

Stimulation of FSH and LH secretion is controlled by gonadotropin-releasing hormone (GnRH), released from the hypothalamus (24, 25). FSH and LH secretion from the pituitary is also modulated by gonadal feedback through steroid and protein factors (26–28). Gonadotropins, FSH and LH, play critical roles in regulating reproduction. In females, FSH induces follicular development, while LH stimulates egg maturation and ovulation in ovaries, and subsequently supports the corpus luteum (29, 30). In males, FSH supports early stages of sperm production in testes and LH stimulates final maturation of sperm through stimulation of testosterone from Leydig cells (31).

Thyrotrophin-releasing hormone (TRH), released from the hypothalamus, regulates TSH secretion with fine tuning by the feedback action of thyroxine (T4) and tri-iodo-thyronine (T3) (32, 33). The primary role of TSH is in stimulating the growth of thyrocytes and biosynthesis of thyroid hormone (T3/T4) through increased uptake of iodide by thyrocytes (34–36). TSHR is also a major autoantigen for autoimmune processes in Grave’s disease (37–41).

Receptors for the glycoprotein hormones belong to the large family of G protein-coupled receptors (GPCRs) that play crucial roles in cellular homeostasis. While GPCRs account for only 3% of the human functional genes, this class of proteins have proven to be extremely valuable as targets for drug discovery with >30% of the small molecule therapeutics developed to date modulating this class of membrane proteins (42–44). Common features shared by GPCRs are their hepta-helical or 7-transmembrane domain (7TM) that links an N-terminal extracellular domain with a C-terminal intracellular domain. The 7TM domain of GPCRs have, in common, three extracellular loops and three intracellular loops that have been shown to be involved in transmission of hormone-binding events into cellular signaling responses (45). GPCRs are activated by variety of stimuli, such as glycoproteins, peptides, neurotransmitters, and ions (46).

**Figure 1:**
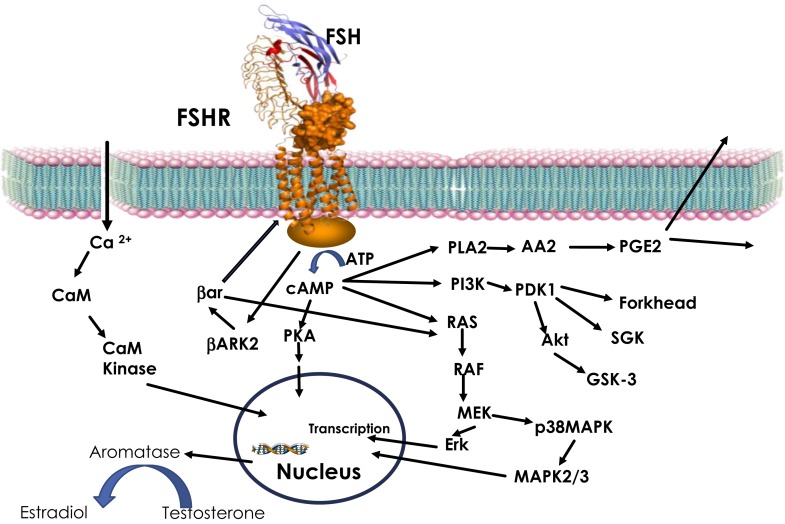
**FSH signaling**. Activation of FSHR by FSH leads to increase in intracellular cAMP through Gs-adenylate cyclase. Increased cAMP leads to PKA activation, which regulate expression of several genes through phosphorylation of transcription factors like CREBP. FSH also causes increase in Ca^2+^ by depolarization of Ca channels. Increased Ca^2+^ can upregulate calmodulin kinase leading to modulation of downstream effectors. In addition to cAMP, FSH has also been shown to modulate PLA, Erk, p38 MAPK, and PI3Kinase pathways. Activated FSHR is phosphorylated by BARK, which in turn recruits β-arrestin to the receptor and lead to down regulation of FSHR, in addition, β-arrestin independently can activate Erk pathway.

The GPCR superfamily can be divided into subfamilies on the basis of phylogenetic analysis of the sequence (47). Glycoprotein hormone receptors belong to the leucine-rich repeat containing GPCR (LGR) subfamily (48). The LGR subfamily is part of the larger Family-A or rhodopsin like GPCR (42, 49). LGRs differ from other Family-A receptors through their extracellular domain. While non-LGRs have a short extracellular region and bind small molecules (e.g., aminergic receptor, opioid receptor, etc.), LGRs have exceptionally large extracellular domains with the leucine-rich repeats (LRRs) of about 340–420 amino acids (50). Binding of glycoprotein hormone to their receptor leads to activation of the receptor by stabilizing the active confirmation (51). Active receptor, in turn, communicates the extracellular event to intracellular signal transducers primarily through a G-protein heterodimer leading to the dissociation of the α and β, γ subunits (52, 53). Following dissociation, the α-subunit stimulates adenylate cyclase, and consequently increases cAMP (54). Increase in intracellular cAMP results in activation of PKA (54). In parallel, the β, γ subunits recruit GPCR-kinases (GRK) to phosphorylate the receptor. This, in turn, leads to the recruitment of β-arrestin to the receptor, resulting in downregulation of the receptor (53, 55–57). In addition to the classical intracellular signal, cAMP, activated glycoprotein hormone receptors have also been shown to invoke other signaling pathways like Ca^2+^, MAPK, and Akt (Figure 1) (54, 58–60). In summary, glycoprotein hormones or FSH, as shown in Figure 1, provokes a complex pattern of gene expression through actions of many different signaling cascades culminating in their physiological response.

### Structure of glycoprotein hormone and their receptor

Structural determination of glycoprotein heterodimers bound to their cognate GPCRs is extremely challenging. However, several groups have utilized improved technological advances in molecular biology, structural biology, and impressive crystallization methods to stabilize and anchor GPCRs to obtain quality crystals. Solving the crystal structure of hCG was a major milestone in the early quest for elucidating the structure for glycoprotein hormones (61, 62). Subsequently, Fox et al. determined the crystal structure of βThr26Ala hFSHR, a partially deglycosylated protein (63). These studies interestingly revealed that both subunits of glycoprotein hormones are folded into elongated non-globular structures belonging to the cysteine-knot superfamily, which includes some growth factors. The heterodimer is stabilized by a segment of the beta subunit, which wraps around the alpha subunit and is covalently linked like a seat belt (61–63). Based on charge distribution, β93–100 (determinant loop), located at the center of the “seatbelt” of beta-hCG conferred specificity of the hormone binding to the receptor. This has been confirmed experimentally by several groups (64–66). In addition to the determinant loop, a second site in the β subunit, L2β has also been implicated in hormone binding to the receptor (67–69). In α-subunit, the CTP 88–92, is required for receptor activation of intracellular signals (70–72). Thus, these three regions of the hormone, the determinant loop, the L2β loop of the β-subunit, and the CTP of the α-subunit, are the major contributors of receptor binding and activation.

Extracellular domain of these receptors can be further divided into two distinct regions, the N-terminal LRR domains, and the hinge region that connects the LRRs to the TMD. In 1990s, several groups identified the importance of the LRR of the extracellular domain of glycoprotein hormone receptor for ligand interaction (73–75). Elucidation of the crystal structure of FSH complexed with truncated FSHR in 2005 by Fan and Hendrickson revealed a detailed interaction of the hormone with the extracellular domain of the receptor (76). The crystal structure revealed that FSH binds to FSHR like “a handclasp” (76). According to their hypothesis, the basal receptor exists as a monomer and ligand binding induces formation of an activated dimer. Recent crystal structure analysis of the complete ectodomain of FSHR confirms that the heterodimeric FSH is bound into the concave surface of LRR in a “handclasp” fashion similar to that described by Fan and Hendrickson (77).

The concave high-affinity hormone-binding surface in the LRR region is a common feature among other members of this family of LRR-GPCRs. TSH and the TSHR stimulating monoclonal antibody M22 bind to the corresponding concave surface of TSHR in the complex (78, 79). Very recently crystal structure of R-spondin with LGR4 and LGR5 revealed that the concave surface of these LGRs is the sole interacting site for R-spondin (50, 80).

**Figure 2:**
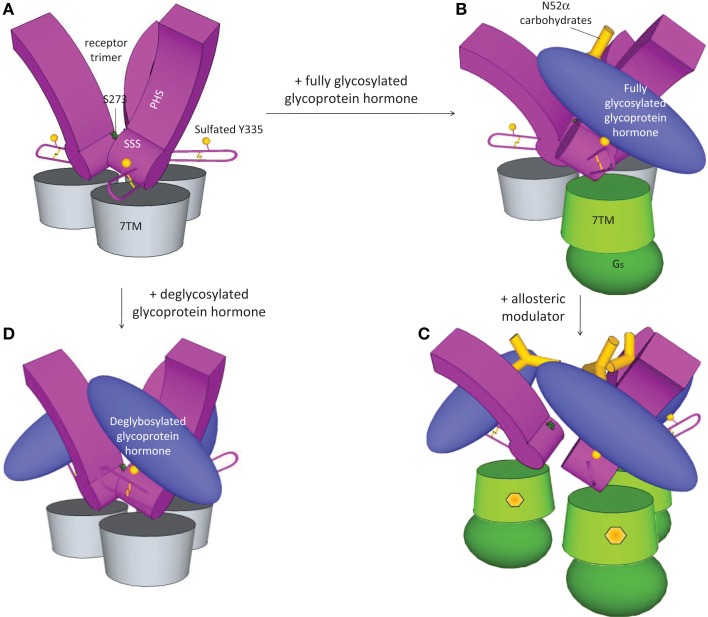
**Activation of FSHR**. Based on the recent crystal structure (77), FSHR exists as trimer in the basal state **(A)**. FSH binding leads to dissociation and activation of the receptor **(B)**. Due to the bulky glycan, only one FSH can bind to the trimeric FSHR. Small molecule modulators by binding to the allosteric site in the transmembrane domain opens up the receptor and enables three FSH binding **(C)**. On the other hand, three deglycosylated FSH can bind to the trimeric receptor without dissociation and activation of the receptor **(D)**.

The role of the hinge domain in hormone binding and signal transduction has been intensively investigated (81–86). Jiang and co-workers identified a critical function for the sulfated Tyr-335 (sTyr) in the FSHR hinge region as a second interaction site with FSH (Figure 2) (77). According to their findings, binding of FSH to the high-affinity inner concave face of the ECD is a transitory event. This first binding event is followed by the formation of sTyr-binding pocket at the interface of α and β subunits of FSH. Then, sTyr is drawn into the pocket lifting the hairpin loop. The lift of the loop releases the inhibitory nature of the hairpin loop and activation of the transmembrane domain. A sulfated tyrosine located in the hinge domain of both LH/CGR and TSHR is also essential for the activation of the hairpin loop domain by their respective ligands (87, 88), suggesting that glycoprotein receptors utilize a common two-step mechanism for ligand recognition and activation.

Glycoprotein hormone receptors have been proposed to undergo dimerization in living cells (86, 89–92). The previous crystal structure of FSH with the extracellular domain of FSHR, lacking the hinge domain, proposed that the dimeric FSH-FSHR may be involved in receptor signaling (76). Evidence supporting intermolecular co-operativity as a component of transactivation of receptor has been cited as supportive evidence for the activated form of the receptor to be a dimer for all three glycoprotein receptors (86, 90, 93, 94). In a very elegant series of genetic models, Huhtaniemi’s group demonstrated that in LHR deficient mice co-expressing equal ratios of both binding deficient and signaling-deficient forms of LHR receptor transactivation can reestablish normal LHR function through intermolecular interaction to restore spermatogenesis (95). In contrast, in transgenic mice expressing only the binding deficient receptor or the signaling-deficient receptor, males were spermatogenically incompetent. Further, single-molecule analysis of these mutant receptors reveal diverse structural assembly of LHR with varying degree of oligomerization that can regulate signaling of the receptor (96).

Zonen and co-workers propose that ligand binding induces strong negative cooperativity within the glycoprotein hormone receptor (86). At physiological concentrations, a single ligand binds a dimer, leading to transmission of the intracellular signal before horizontally causing a negative impact on the transmembrane domain of the other protomer. This leads to lowering of the binding affinity of the second protomer, providing allosteric cooperativity across the receptors (86). Very recently, single-molecule analysis of FSHR/LHR co-expressed in HK293 cells demonstrates heterodimeric interaction between FSHR and LHR (97). This heterodimeric interaction results in attenuation of LH-induced signaling through LHR and attenuation of FSH-mediated signaling through FSHR. The authors propose that heterodimerization of glycoprotein receptors could play a significant role in fine tuning the signaling event of FSH and LH during granulosa cell differentiation. However, it will be critical to demonstrate heterodimerization in primary cell systems and in *in vivo* models where the receptor expression is at low level compared to the overexpression system.

The recent crystal structure of the FSH complexed with the complete extracellular domain of FSHR challenged the previous view of the structural changes imposed on this receptor upon ligand binding (98). According to this model, in basal state, FSHR exists as a trimer (Figure 2A), and only a single unit of fully glycosylated FSH bind the trimeric receptor (Figure 2B), leading to dissociation and activation of the ligand-bound monomeric receptor. On the other hand, due to the lack of bulky glycans, three deglycosylated hormones can bind to the receptor keeping it in the trimeric inactive state (Figure 2D). Although the trimer model of FSHR in FSH recognition could well explain some observation in biochemical and functional studies, the *in vivo* relevance of the FSHR-FSH trimerization and the actual oligomerization form in living cells still need to be determined.

### Small molecule modulators of glycoprotein hormone receptors

Development of drugs that target the ligand-binding domain has been highly successful for agonists or antagonists that address the large superfamily of GPCRs. Unfortunately, many of the current GPCR-based drugs produce unwanted dose-limiting side effects due to cross reactivity with other related receptors that share structurally conserved features. Yet, another challenge for developing innovative drugs targeting GPCRs is that many of the synthetic molecules that replace peptide or protein ligands have been intractable (not “drug-able”) largely because the molecules must fit into highly lipophylic regions of the GPCR transmembrane domains (99). However, for the past several decades, it has been realized that receptors can be regulated by allosteric sites that are distinct from the ligand-binding orthosteric site (100). Accordingly, there is now ample evidence over the past decade and half that a GPCR response to endogenous ligand can be modulated by synthetic small molecules targeting allosteric sites (101–105). These allosteric modulators can exert negative or positive effects on endogenous ligand signaling. There are four types of allosteric ligands, antagonist known as negative allosteric modulators (NAMs), potentiators also called positive allosteric modulators (PAMs), allosteric agonists (allo-agonists), and finally silent modulators (SAMs) (106). For glycoprotein hormone receptors, since the ligands are very large and involve multiple binding sites at the receptor, a small molecule binding the orthosteric site cannot be envisaged. The advent of allosteric modulators in other GPCR programs has encouraged the incorporation of drug discovery strategies to screen for allosteric modulators that modulate glycoprotein hormone receptors.

The primary market driver invoked by drug discoverers to pursue allosteric modulators for glycoprotein hormones over available injectable proteins is patient convenience. A secondary motivation is the hope that a new mechanism of action for allosteric agonists can improve the biological response relative to “glyco-uniform” biotherapeutics. As the number and quality of allosteric modulators increases, subtle advantages of PAMs or NAMs over the injectable proteins are beginning to emerge. In the late 1990s, recombinant therapeutic proteins were developed to provide superior consistency than could be obtained by purification of hormones from natural sources. However, the recombinant proteins continue to be administered by injections, which are inconvenient and results in low patient engagement for infertility treatment. Recombinant proteins are also constrained by regulatory requirements for uniform post-translational modifications, such as glycosylation. The preference of patients and physicians for orally active therapeutics has motivated development of replacements for injectable treatments in rheumatoid arthritis (anti-TNF agents vs JAK inhibitors) (107, 108) and multiple sclerosis (interferons vs Fingolimod or Teriflunomide) (109, 110). For infertility patients, the motivation is similar; it is more desirable to have small molecule agonists of glycoprotein hormones that can be used as an oral therapy. Secondarily, if the allosteric agonist can amplify the receptor response to endogenous biodiverse glycoprotein forms, this may provide a preferred therapeutic over suppressing endogenous glycoprotein production followed by replacement with a bio-constrained uniformly glycosylated glycoprotein. A NAM of FSHR and/or LHR may lead to development of a highly specific oral contraceptive with lesser side effects than the currently available steroidal-based drugs. In addition, glycoprotein hormone receptor small molecule antagonists may have a better long-term safety than steroidal contraceptives (111–113).

Developing small molecule agonists or antagonists for glycoprotein hormone receptors has been challenging for medicinal chemists; however, in the last few years, great strides have been achieved in developing chemical scaffolds targeting the glycoprotein receptors through advances in screening tools, access to larger diverse library of small molecule compounds and robotic systems to conduct high-throughput campaign. Most of the new allosteric modulators have been identified through high-throughput screening (HTS) campaigns using cell-based assays (114–117). The availability of a wide range of assays from overexpressed isolated proteins to engineered cell culture systems measuring second messengers, to primary cell cultures, and to *ex vivo* animal tissues has made it critical to identify the appropriate system for screening as well as various transitions to more physiologically relevant models. The key objective of the optimal screen is to quickly filter false positives identified because of the artificial system and confirm their activity in physiologic cellular responses that address the therapeutic goal (118).

In our own drug discovery experience, there has been an evolution in the approaches we have used to identify and develop small molecule agonists of glycoprotein hormone receptors. In the process, we learned three key lessons: (a) molecules that stimulate cAMP in immortalized cell systems expressing FSH receptor (FSHR) as a primary screen do not necessarily reflect the compound requirements to stimulate follicular development; (b) the diversity of intracellular and intrafollicular events stimulated by FSH cannot be reproduced by measuring single endpoints in single cell types *in vitro;* and (c) the highest potency compound *in vitro* does not always correlate with best efficacy *in vivo*. In the next several sections, we will highlight how these lessons influenced our current discovery process.

#### Molecules that Stimulate cAMP in Immortalized Cell Systems Expressing FSH Receptor as a Primary Screen Do Not Necessarily Reflect the Compound Requirements to Stimulate Follicular Development

It is intuitively obvious that it is a huge challenge to engineer a small molecule (molecular weight 500–600) that can replicate the integrated biochemical response of a large protein (molecular weight 33,000). A small molecule glycoprotein hormone receptor agonist cannot occupy the same space in the extracellular ligand-binding domain of the FSHR. Therefore, binding assays were discarded as a primary screen in these programs, but instead were applied to understand changes in receptor conformations induced by the small molecule. Cell-based assays using physiologically reasonable levels of expression of the appropriate receptor and intracellular signaling cascades are important to interpret screening results. Exaggerated overexpression systems can make a weakly active compound look more potent than it really is. FSHR expression in our CHO-cell system, as detected by FSH binding to receptors, was approximately threefold greater than expression in primary granulosa cells. It has been demonstrated that receptor density at the plasma membrane can control the balance between distinct signal transduction pathways (56).

#### Diversity of Intracellular and Intrafollicular Events Stimulated by FSH Cannot be Reproduced by Measuring Single Cell, Single Outputs in Cell Culture

It is imperative to confirm changes in second messengers induced by allosteric modulators, in subsequent primary cell culture systems that measure physiologically relevant products associated with the same intracellular pathways. In the earliest attempts to identify FSHR agonists, compounds with activity in FSHR-expressing CHO cells were quickly advanced into *in vivo* models without evaluation in primary granulosa cells. Over several iterations of screening, we learnt that the structure–activity relationship (SAR) from immortalized CHO cells does not translate well to activity in rat granulosa cells or human granulosa cells. Compounds active in the immortalized cell screen were tested in relevant functional assays using rat granulosa cell cultures and measuring estradiol secreted in the media. Responses of the compounds varied between CHO-hFSHR cells and in granulosa cells (Table 1). Among compounds that were moderately potent in CHO cells (EC50 between 10–49.9 nM and 50–250 nM) ~30% of compounds were ineffective in stimulating estradiol in the functional assay (Table 1). Furthermore, among compounds that were potent in CHO-FSHR cells (1–4.9 nM and 5–9.9 nM EC50), ~25% of the compounds were unable to stimulate estradiol production in granulosa cell culture. Among the most potent compounds (EC50 < 1 nM), only 7% of compounds were inactive in granulosa cells (Table 1). This data suggests that the result obtained from overexpression system should be treated with caution, as response in functional assay at the beginning of the lead optimization effort had nearly 30% false positive rate.

**Table 1:** **Not all compounds active in CHO-hFSR cells can stimulate estradiol secretion in primary rat granulosa cells**.

EC50 in ­CHO-hFSHR (nM)	Rat granulosa cell assay (GC)		
	No. of compounds		
	Tested in GC	No activity	% Inactive
<1	44	3	7
1–4.99	97	20	21
5–9.99	52	15	29
10–49.9	84	30	36
50–250	10	3	30

The converse relationship between physiological activity in granulosa cells and signaling activity in immortalized cells was observed for FSHR NAMs. An FSHR NAM was shown capable to partially reduce cAMP induced by FSH in HEK293 cells, but it was very effective in blocking cAMP and progesterone production in a primary granulosa cell system (119). This clearly highlights that the SAR developed in an immortalized cell system is not always transferable to the physiologically relevant functional cell model. It is imperative to test molecules in a therapeutically relevant cell very early on in the screening program before progressing molecules to animal models.

#### Highest Potency Compound *In Vitro* Does Not Always Correlate with Best Efficacy *In Vivo*

The correlation between granulosa cell activity and *in vivo* activity is much lower, and is affected by multiple variables. Compounds in our program as well as compounds from other efforts [thiazolidinones (TZDs)] that are very potent (EC50 < 1 nM) are poorly absorbed and/or extensively metabolized following oral exposure (117, 120). In general, these molecules have very high log*D* values, and are metabolized faster. One has to balance the desire for highly potent compounds with candidates that can be orally available. Highly potent compounds frequently share undesirable absorption, distribution, and metabolism (ADME) properties. There are some excellent reviews published on the small molecule allosteric modulators of glycoprotein hormone receptors (121–123). We will focus on the most recent advances made in this exciting field.

### FSH receptor modulators

**Figure 3:**
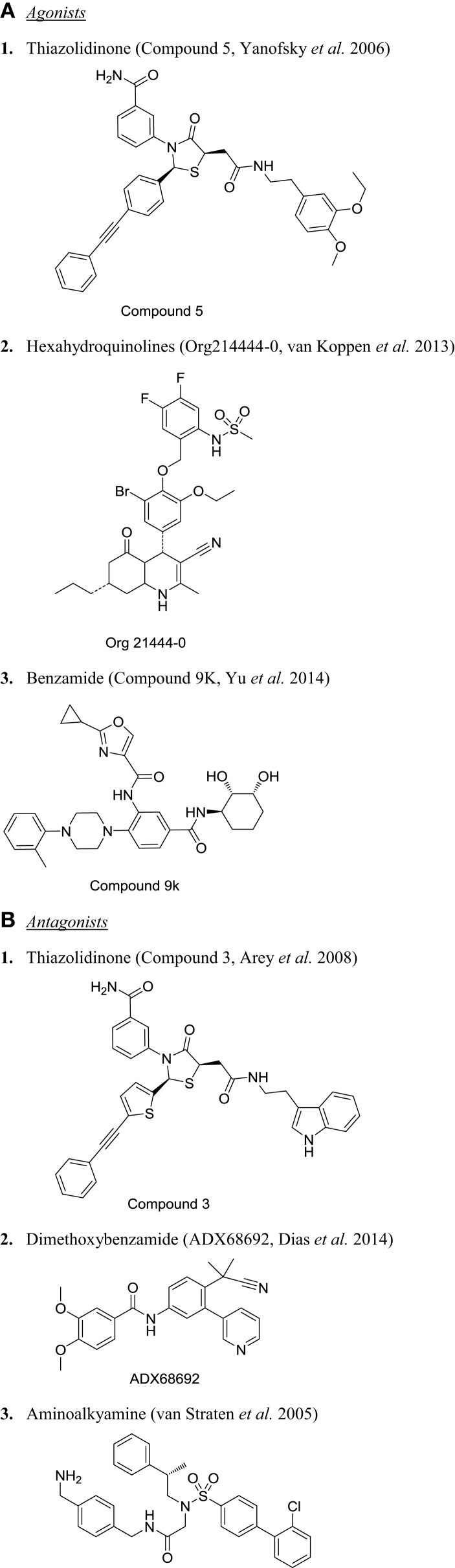
**Chemical structure of selected small molecule modulators of FSHR**. **(A)** Agonists: (1) thiazolidinone [compound 5 (127)], (2) hexahydroquinolines [Org214444-0 (135)], and (3) benzamide [compound 9K (117)]. **(B)** Antagonists: (1) thiazolidinone [compound 3 (125)], (2) dimethoxybenzamide [ADX68692 (147)], and (3) aminoalkyamine (132).

Among the three glycoprotein hormones used in infertility treatment, FSH is the major value driver for therapeutic intervention. Without the FSH treatment, there is no ovarian hyperstimulation. As expected, there are several publications on FSHR modulators and fewer reports on development of LHR and TSHR modulators. The first report of FSHR agonist was published as a patent in 2001 by Serono (124), describing a piperidine carboxyamide, which had an EC50 of 3.9 nM in CHO-hFSHR cells measuring cAMP. These molecules were originally identified through HTS of a compound library. Unfortunately, piperidine carboximides lacked *in vivo* activity. In this program, there was virtually no systematic structure-based optimization of the lead through iterative Med Chem efforts using granulosa cell cultures to guide their development. Since then, several groups have followed up with various chemical scaffolds targeting FSHR, including TZDs (125–127), substituted gamma-lactam (128), diketopiperazines (129, 130), N-alkylated sulfonyl piperazine (131), tetrahydroquinolines (132), hexahydroquinoline (133), thienopyrimidines (134), and benzamides (117). Chemical structures for some of these are provided in Figure 3. For more detailed review on chemical nature of other series, please refer to van Straten and Timmers (123). The cellular and physiological effects of specific chemical classes are summarized below.

#### Thiazolidinones Agonists

Thiazolidinones were identified through a combinatorial library screening (126–128). The initial hit obtained through screening had an EC50 of 20 μM in CHO-hFSHR cells, but this potency was optimized over 10,000-fold during lead optimization (127). In addition to their effects in immortalized cells, TZDs were capable of stimulating estradiol production in functional rat granulosa cells (127). These authors further explored the site within the receptor where these compounds might be working. Using multiple reconstitutions of FSHR and TSHR transmembrane domain chimeras, they identified that TZDs activated FSHR through the transmembrane domain 1–3 (127). They also observed a range of biochemical features of this series of compounds from PAM to mixed modulators and to negative modulators (125), suggesting that a small change in the TZD scaffold can provide FSHR analogs of differing pharmacology. The agonists stimulated cAMP and estradiol in granulosa cells. On the other hand, the negative modulators were completely devoid of agonistic activity and inhibited FSH-induced cAMP and steroidogenesis, through activation of Gi pathway. The mixed modulators at lower concentrations behaved as agonist stimulating cAMP through Gs, while at higher concentration, the compound activated Gi pathway and reduced cAMP demonstrating negative cooperativity as was demonstrated within cells expressing constitutively active glycoprotein receptors (86, 125). These molecules provide evidence that it is possible to selectively trigger specific signaling pathways of the receptor. Recently, our group demonstrated that the TZD compound was capable of stimulating multiple signaling pathways, in addition to cAMP, in an integrated cellular signal very similar to FSH (120). These molecules behaved as “allo-agonists,” in that they can potentiate FSH action in the presence of low FSH, and also directly activate FSHR in rat granulosa cells (120). In relevant physiological cellular models, TZD stimulated cumulus expansion of granulosa cells, and induced *in vitro* follicular growth. Due to extremely low oral bioavailability, TZD was not suitable as orally active therapeutics; however, the compound was quite effective in stimulating follicular development in immature rat when delivered continuously by Alzet pump (120).

#### Hexahydroquinoline Agonists

A series of hexahydroquinolines with nanomolar activity in CHO cells were reported (133). Cyclocondensation reaction of hexahydroquinolines resulted in mixtures of four diastereoisomers with EC50 <1 nM. One such compound, Org214444-0, was highly lipophilic and stereoselective on FSHR over related LHR and TSHR (135). Org214444-0 was quite potent in stimulating rat and human granulosa cells. In binding experiment with ^125^I-FSH, this compound was able to increase FSH binding affinity by 6.5-fold, while in CHO-CRE luciferase assay, a three to fivefold increase in potency was observed, behaving as “allo agonist.” They also demonstrated oral bioactivity as measured by increased follicular development and ovulation in mature rats (135). This is the first report of an orally active FSHR molecule. In an attempt to reduce the lipophilicity, several pyridyl- and sulfonamide-substituted hexahydroquinolines were prepared (136). The compounds had moderate *in vitro* activity but there is no report on their *in vivo* potency.

#### FSHR Antagonists

In addition to the pursuit of the development of small molecule FSHR agonists to promote fertility, several novel series of compounds have shown potential to suppress fertility as contraceptives. Contraceptives have played a significant role in avoiding unwanted pregnancy, for family planning, and for slowing population growth. Currently the widely used contraceptives are steroid based and have a number of side effects, so developing a safer method of contraceptive is a significant unmet medical need (137, 138). FSH plays a critical role in follicular development and the onset of sperm production (139–142). Thus, blocking FSH action with a receptor antagonist can be a novel non-steroidal-based approach with specific activity in ovary and testes without affecting other peripheral and central tissue.

The first report of an FSHR antagonist for use in contraception was reported in 2002 (143). The compound inhibited FSH-induced cAMP and steroid production. *In vivo* at 100 mg/kg, provided by ip, the compound blocked increases in ovarian weight and ovulation (143). Aminoalkylamides were described in 2003 to have antagonistic activity against FSHR (144). Two compounds were tested for their ability to interrupt estrous cycle in female rats and their effect on spermatogenesis in male, but in both cases, the compounds were not very effective (144). Organon later reported identification of tetrahydroquinolines agonists from HTS with EC50 on FSHR at 4.4 μM (132). Hit optimization of this series led to switch from agonist to antagonist with IC50 of 5 nM (132). This compound showed antagonistic activity in granulosa cells and inhibited *in vitro* follicle growth and ovulation (132). However, *in vivo* efficacy of this molecule was not reported. Antagonists with greater potency were obtained with dimeric compounds (145). Connection of two weakly antagonistic molecules with a spacer of sufficient length generated antagonists with much better activity *in vitro* (145).

Small molecule FSHR agonists have varying pharmacokinetics properties, hence shown to have quite different half-life. A clever approach was used by van de Lagemaat to develop a contraceptive using a FSHR agonist with a very short half-life (146). Follicle stimulation in mammals is achieved when the circulating concentration of FSH is sustained above the threshold for sufficiently long time. Thus in rats, optimal follicular growth occurs only when FSH is administered twice a day for 2 days due to its short half-life, in contrast to a single injection of PMSG, which has longer half-life and remains in the circulation for a longer duration (119). Van de Lagemaat et al. observed that oral administration of the short-acting FSHR agonist inhibited ovulation by inducing premature luteinization of unruptured follicles in rat and guinea pig (146). This effect was reversible; therefore, this novel approach of short follicular stimulation followed by premature withdrawal presents a unique mechanism of contraceptive action relative to that used by steroidal hormones, which blocks the entire ovarian follicular phase. However, in cynomolgus monkey, the effect of the compound was partial as only about 40% of animals showed luteinized unruptured follicle. Due to the variation in response in non-human primate, this molecule was not pursued for development, but the approach is quite novel. Thus, having allosteric modulators with differing pharmacology can be a useful tool for both stimulating and controlling fertility.

Investigators from Addex, in collaboration with Dias and co-workers, characterized three of the NAM identified from their drug discovery effort. These are low molecular weight compounds effective in blocking FSH-induced cAMP production in CHO-hFSHR and rat granulosa cells. In the first paper, they demonstrate ADX61623 to increase the affinity of ^125^I-hFSH binding to the receptor (119). In rat granulosa cells, FSH-induced progesterone secretion was inhibited by ADX61623, but not estradiol, demonstrating biased antagonism on FSH signaling. This molecule was only partially effective in blocking FSH-induced follicular development and ovulation in rats (119). Results with ADX61623 provide proof that small molecule modulator of FSHR can be used to dissect the signaling pathways of the receptor. In a more recent publication, Dias et al. have tested two other NAMs, ADX68692 and its analog ADX68693 (147). ADX68692 inhibited FSH-induced progesterone and estradiol production in granulosa cells. *In vivo*, this compound blocked FSH-mediated follicular maturation and ovulation in immature rats (147). While in mature cycling rats, though ADX68692 disrupted estrous cycle, it had only a partial effect in blocking pregnancy following mating (147). Its contraceptive efficacy in mature rat remained lower than that can be achieved with steroidal contraceptives. ADX68693, on the other hand, showed biased antagonistic activity on FSH-mediated steroid production like ADX61623 by inhibiting FSH-stimulated progesterone, but rather stimulating estradiol secretion in granulosa cells and no significant effect in blocking ovulation in immature rat (147). These studies demonstrate that, for an effective contraception, it is critical to inhibit both arms of FSH-induced steroidogenesis, i.e., progesterone and estradiol biosynthesis. At present, the available FSH antagonists lack pharmacological properties that would justify development as alternatives to steroidal contraceptives.

### LH receptor modulators

The first series of small molecule LHR agonist reported in literature were thienopyrimidines (116). An HTS campaign followed by hit optimization resulted in Org41841 with EC50 of 20 nM in CHO-hLHR assay. This compound stimulated testosterone in mouse Leydig cells. Org41841 at 50 mg/kg administered orally induced ovulation in 40% of immature mice primed with FSH (116). This was the first report of oral activity of LHR analog. Intensive medchem optimization of thienopyrimidines led to the identification of several potent molecules. One compound, a trifluoroacetic acid salt form of thienopyrimidine, Org42599 behaved as a pharmacochaperone of mutant LHR (148). In previous work, mutations in LH receptors (LHRs) at two locations, A593P and S616Y, cause misfolding of the receptor and these receptors fail to get trafficked to the plasma membrane. Org42599 facilitated expression of the mutant receptors to the plasma membrane behaving as pharmacochaperone and rescued the stimulatory response to LH (148). This approach may have translational application for treatment of patients bearing such mutation.

A series of pyrazole compounds with mixed FSH/LH activity was reported (149). Compound 5 was described to have an EC50 of 20 nM (efficacy 53%) in CHO-hLHR and an EC50 value of 130 nM (efficacy 73%) in CH-hFSHR assays (122). This compound stimulated testosterone production in rat (122, 149), though its effect on follicular development was not reported. Bonger et al. obtained a highly selective LHR agonist by linking a dual LHR/FSHR molecule to a previously characterized FSHR antagonist (115, 150).

Another interesting molecule from optimization of thienopyrimidine is Org43553. This molecule stimulated LHR to produce cAMP with EC50 3.4 nM, while FSHR was activated at 110 nM (151). LH at higher concentration can activate phospholipase C (PLC), but Org43553 inhibited LH-induced PLC, showing biased agonism on LHR (152). Pharmacokinetic analysis showed that the compound had high oral bioavailability with short half-life (151). Oral administration of Org43553 induced ovulation in female (immature mice and adult rat) and serum testosterone in male rat (151). Since the half-life of this molecule was shorter than that of hCG, it can potentially reduce the risk of ovarian hyperstimulation syndrome (OHSS), a condition believed to be induced by hCG (153–155). In fact, in rat, Org43553 induced ovulation without the increased vascular permeability or increased expression of vascular endothelial growth factor (VEGF), caused by hCG (156). At present, it is unclear if it is biased agonism or short half-life of the Org43553 that is able to induce ovulation, but reduce the risk of OHSS (156). Finally, in the most exciting study, Org43553 and another molecule Org43902 were well tolerated in normal healthy women and demonstrated that single oral administration of the small molecule agonist induced ovulation of gonadotropin-stimulated, mature follicles in pituitary-suppressed women (157). This represents a giant stride toward the demonstration of proof of concept for development of an orally active small molecule modulator of glycoprotein hormone receptor in human.

#### Antagonist

**Figure 4:**
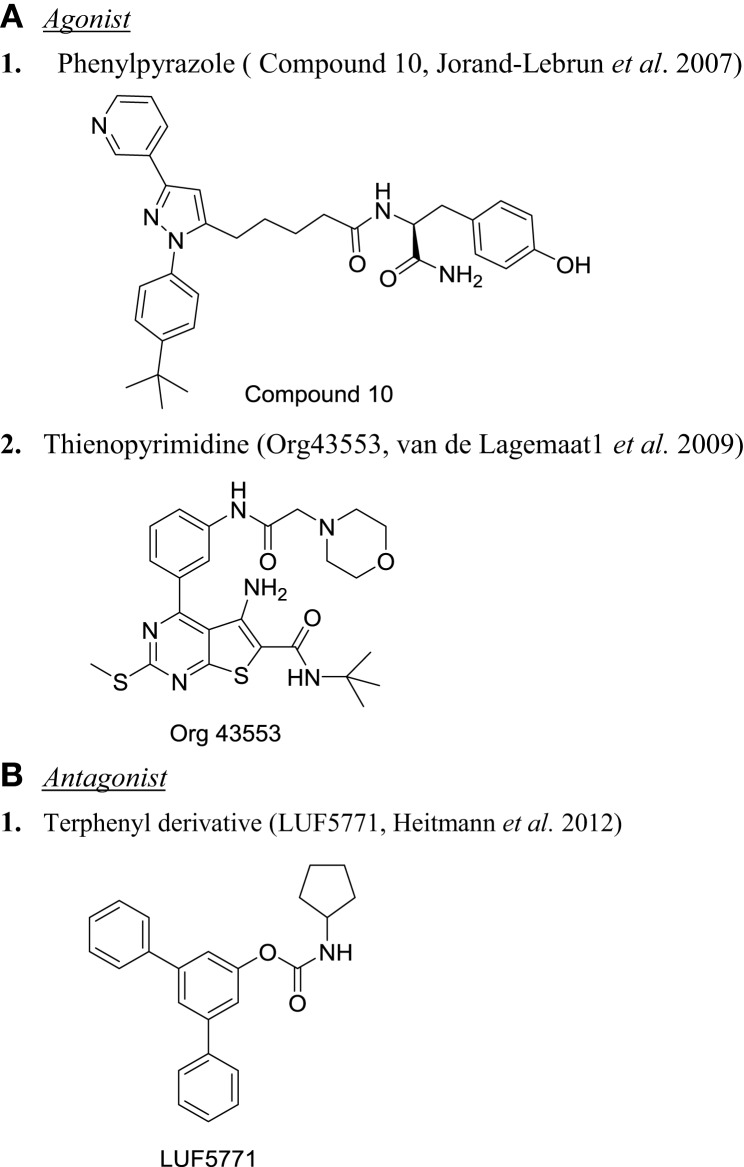
**Chemical structure of LHR modulators**. **(A)** Agonist: (1) phenylpyrazole [compound 10 (149)] and (2) thienopyrimidine [Org43553 (151)]. **(B)** Antagonist: (1) terphenyl derivative [LUF5771 (158)].

Development of LHR antagonists is very limited. A binding assay using small molecule radioligand ^3^H-Org43553 (LHR agonist, described above) was used as a screening tool to identify LHR antagonists (158). Binding of ^3^H-Org43553 with hLHR membrane was saturable with *K*_d_ of 2.4 nM and *B*_max_ of 1.6 pmol/mg protein (159). Five small molecule agonists evaluated in this assay showed good correlation between binding affinity relative to Org43553 and their potency in cellular assay (159). Using this binding assay as the screening tool, terphenyl derivatives were identified to inhibit ^3^H-Org43553 binding to the membrane (158). Interestingly, one of the derivatives, compound 24 (LUF5771) was able to increase the *K*_d_ of ^3^H-Org43553 by 3.3-fold. In a functional assay, LUF5771 inhibited the activation of the receptor by hLH and Org43553. *In vivo* efficacy of LUF5771 as an allosteric inhibitor was not demonstrated. Figure 4 illustrates the chemical structure of some of the interesting LHR modulators.

### TSH receptor modulators

**Figure 5:**
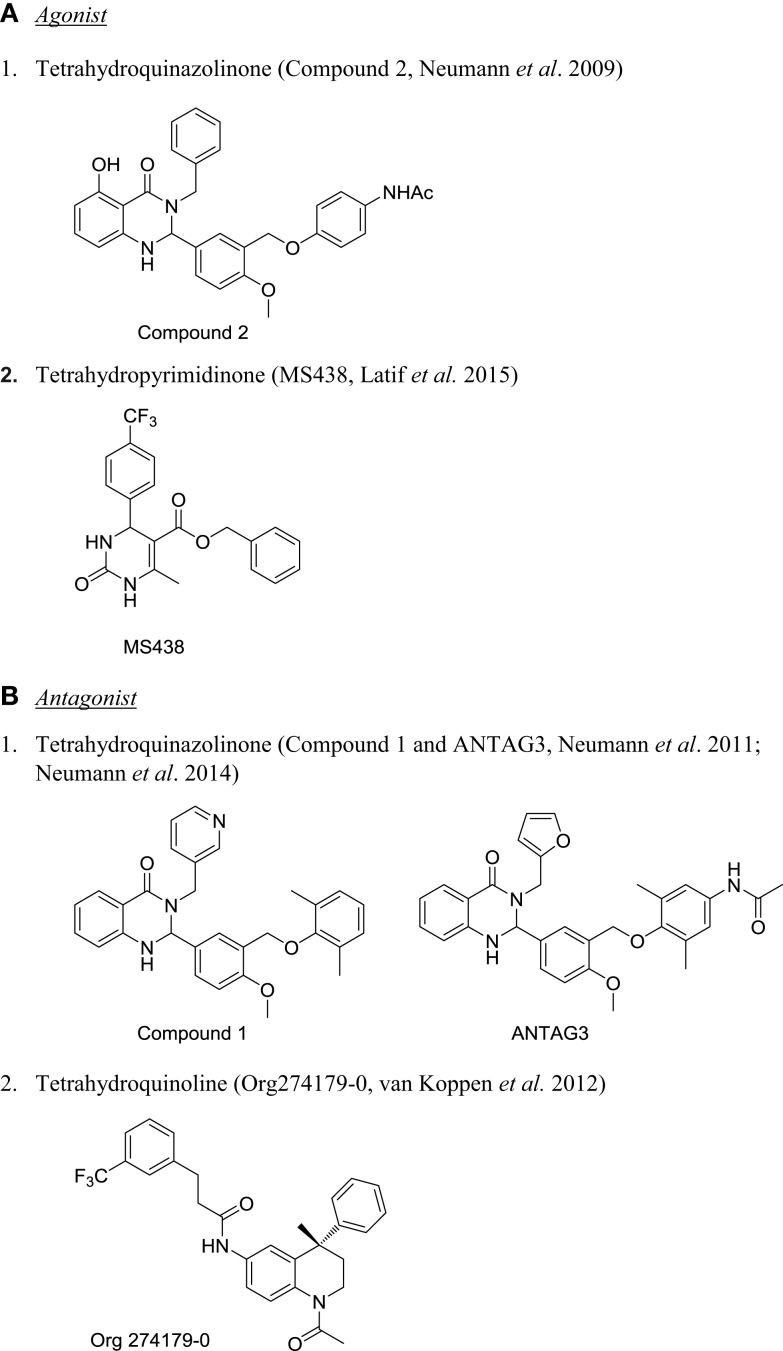
**Chemical structure of TSHR modulators**. **(A)** Agonist: (1) tetrahydroquinazolinone [compound 2 (163)] and (2) tetrahydropyrimidinone [MS438 (114)]. **(B)** Antagonist: (1) tetrahydroquinazolinone [compound 1 and ANTAG3 (165, 169)] and (2) tetrahydroquinoline [Org274179-0 (167)].

For the past few years, there are reports on the development of small molecule allosteric modulators for TSHR (160, 161). The chemical structure of few of the TSHR allosteric modulators are shown in Figure 5. The first TSHR agonist started from a thienopyrimidine, Org41841, the LHR agonist (116). Due to high homology between TMD of LHR and TSHR, it was predicted that Org41841 would bind to TSHR. This prediction was confirmed by docking studies, and eventually experimental results identified Org41841 as a partial agonist (162). Further, HTS and optimization of a hit resulted in identification of compound 2 (C2), which was a full agonist at TSHR with an EC50 of 40 nM and no activity at FSHR or LHR (163). More importantly, C2 was active in a physiologically relevant cell system, primary human thyrocyte culture. C2 stimulated serum thyroxine (T4) in mice when administered orally (163). This is the first proof of principle study that a small molecule agonist for TSHR is active in an *in vivo* preclinical model. However, there is no report on further development of the compound. Very recently, Latif et al. described two molecules, MS437 and MS438, with potent activity on TSHR and thyrocytes (114). Both these molecules demonstrated *in vivo* activity in stimulating thyroxine in male rats when administered intraperitoneally. It is not known if these molecules are orally active. Molecular docking studies showed that these compounds bind to the TMD3 of TSHR (114).

The first antagonist against TSHR also had its origin from Org41841. Based on the model of Org41841–TSHR complex, it was predicted that elongated analogs would bind differently to the receptor, so a long propylene-methyl-ether group was added at the para position of the aromatic moiety to obtain compound 52 (164). This compound inhibited TSH-stimulated cAMP by 71% with an IC50 of 4.2 μM and blocked antibody-induced thyroperoxidase mRNA in primary thyrocytes, suggesting it could be used in pathological condition like Grave’s disease (164). However, due to its weak potency, it could only be used as a starting point to make more potent analogs to develop as therapeutics. This group also described developing a TSHR inverse agonist with antagonistic activity that can block TSHR antibody-induced orbital fibroblast functions (165, 166). Van Koppen et al. reported development of Org274179-0 as nanomolar potent allosteric antagonist capable of inhibiting M22-induced cAMP production in orbital fibroblast, but this compound was equally effective in inhibiting FSHR as well (167, 168). Perhaps, the most potent and selective TSHR antagonist, ANTAG3, inhibited TSH and M22-induced elevation of serum-free T4 and mRNAs for thyroperoxidase and sodium-iodide cotransporter *in vivo* (169). Availability of several chemical series of small molecule modulators of TSHR opens an exciting opportunity for developing novel therapy for pathological conditions as well as to use them as research tools to understand the basic biology of TSHR signaling.

### Mechanism of action of small molecule modulators

Small molecule modulators of glycoprotein hormone identified to date, except for stilbene bisulfonic acid 20 (143), do not displace the binding of the protein ligand. Several chemotypes have been demonstrated experimentally to bind to the transmembrane domains of the receptors (127, 162, 170). Molecular modeling and mutagenesis studies have helped us to understand the functional mechanism of allosterism of small molecule modulators of the glycoprotein receptors. The structure of rhodopsin is adopted as a general model for elucidating the functional domains of all GPCRs. Using this approach, it is proposed that there are two pockets of allosteric binding sites in the transmembrane regions of glycoprotein receptors (121, 170). Pocket1 (P1) is formed between TMD III, IV, V, and VI, while the 2nd pocket (P2) is formed by TMD I, II, III, and VII (121, 170, 171). P1 has been suggested to be the site of interaction of pyrazole, pyrimidine, and tetrahydroquinoline chemotypes (121, 162). Using chimeric FSHR–TSHR hybrid receptors, activity of TZD on FSHR was dependent on presence of transmembrane regions I, II, and III, suggesting this chemical series interacts with the P2 pocket of the receptor (121, 127).

Based on the docking studies and experimental evidence with Org43553 and two other small molecules (LUF5771 and LUF5419), Heitman et al. confirmed two binding sites in the transmembrane region of LHR for small molecule modulators (170). By *in silico* docking studies, the binding site for LUF5771 was proposed to be in the pocket created by TMDs 1, 2, 3, 6, and extra cellular loop 2, corresponding to site P2 [reviewed in Ref. (121)]. Org43553 interaction was restricted to site P1 (170). LUF5771, the allosteric inhibitor, strongly overlapped with the binding site of LUF5419, an allosteric enhancer of Org43553. However, the antagonist interacts with additional residues in TM2 and 7, which are likely to restrict the receptor in an inactive conformation (170). It is noteworthy to mention that similar structural constraint is induced by compound 52 on TSHR to behave as an antagonist (161). The existence of multiple allosteric sites on glycoprotein hormone receptors provides opportunities to design and develop new compounds with improved selectivity and therapeutic value.

Recent crystal structure of FSH with FSHR extracellular domain provided evidence for the existence of trimer in the basal state (Figure 2A) (98). Receptor trimerization is mediated by both the transmembrane domains and the ectodomain. Binding of one fully glycosylated FSH to the basal trimer results in dissociation of a single monomer from the trimer, resulting in the activation of the single monomer (Figure 2B). Based on this model of hormone receptor activation, refined through the use of several small molecule ligands, the mechanism proposed is that small molecules induce 1–3 active monomers in concentration-dependent manner that can be monitored by binding of three glycosylated FSH heterodimers to the dissociated monomers (Figure 2C) (98). Increased binding of FSH in the presence of the modulators confirms this observation (119, 135, 172). An important question is whether the FSHR trimer described by the new crystal structure is functionally relevant in physiological systems.

## Summary

With greater understanding of GPCR biology and improved methods to develop allosteric modulators, several chemical series have emerged. Currently, we are pursuing a new chemical series for FSHR modulator, which has shown great promise in preclinical models including DMPK, safety, and toxicology studies. Successful development of an oral LH/hCG as well as GnRH antagonist modulator in clinical studies has kindled our hopes to have an oral therapy for fertility treatment in assisted reproductive technology. Encouraging progress in the development of allosteric modulators of GPCRs can transform the dream of physicians and patients for having a more convenient therapy associated with infertility treatment into reality. Access to an orally active glycoprotein hormone agonist provides hope for patients that are considering dropping out of the treatment due to the stress involved in injection of the drugs. Similarly, for contraceptive development, a new chemical series of antagonists have emerged, which are quite encouraging for future. The availability of small molecule ligands for TSHR would widen the therapeutic interventions for thyroid cancer and patients with hyperthyroidism. Both agonists and antagonists of glycoprotein hormones will be useful as pharmacological tools to conduct further basic and applied research in understanding the molecular regulation in hormone binding, signal transduction, and biased signaling. The future holds special promise for development of novel oral allosteric modulators of glycoprotein hormone receptors as the third generation therapeutics after purified and recombinant hormones.

## Conflict of Interest Statement

Selvaraj G. Nataraja, Henry N. Yu, and Stephen S. Palmer are founders and employees of TocopheRx; Henry N. Yu is also an employee of EMD Serono Research and Development Institute Inc.
